# Advances in influence factors of ultrasound-guided percutaneous thermal ablations for benign thyroid nodules: A review

**DOI:** 10.1097/MD.0000000000039218

**Published:** 2024-08-09

**Authors:** Liying Wang, Shusen Zheng

**Affiliations:** a Department of Ultrasound, Shaoxing Second Hospital, Shaoxing, Zhejiang, China; b Key Laboratory of Combined Multi-Organ Transplantation, Ministry of Public Health, Zhejiang University, Key Laboratory of Organ Transplantation, Hangzhou, Zhejiang, China.

**Keywords:** influence factor, minimally invasive, thermal ablation, thyroid nodules, ultrasound intervention

## Abstract

Ultrasound-guided percutaneous thermal ablation is a safe and effective minimally invasive treatment for benign thyroid nodules, which is now widely used in the world. Studies have found that some preoperative factors played an important role in the outcome of thermal ablation. This paper mainly reviews the various factors affecting the efficacy of ultrasound-guided percutaneous thermal ablation in the treatment of benign thyroid nodules to provide a variety of perspectives for the clinical and to promote the postoperative outcome of patients.

## 1. Introduction

Thyroid nodules (TN) are abnormal structures in the thyroid gland caused by many factors such as heredity, environment, living habits, emotion, etc. They are the most common disease of the thyroid gland. With the development of high-frequency ultrasound technology, the detection rates of thyroid nodules have increased year by year. Ninety percent to 95% of the nodules are benign, and their clinical manifestations are asymptomatic with no need of treatment. However, some benign nodules need clinical treatment because they show progressive growth, and can cause local compression and discomfort, even excessive anxiety because of cosmetic changes.^[1–3]^ At present, the common treatment for benign thyroid nodules is traditional surgical resection, which can relieve the symptoms of patients and reduce the possibility of malignant transformation. However, since there are reductions of normal thyroid glands after surgery, the thyroid hormone secreted by the thyroid glands is reduced, resulting in hypothyroidism. The patients need thyroid hormone replacement treatment for life, while long-term use of thyroid hormone may cause osteoporosis, atrial fibrillation and other adverse reactions. In addition, laryngeal nerve injury, bleeding, surgical scar, postoperative adhesion, hypoparathyroidism and other complications can also occur.^[4]^

In 2006, Kim et al^[5]^ first reported that thermal ablation had positive effects on benign thyroid nodules. According to the researches of the National Library of Medicine database, ultrasound-guided laser thermal ablation of thyroid nodules is the most effective minimally invasive treatment of benign thyroid nodules, and is an alternative to surgical intervention. As a result, thermal ablation is gradually emerging, becoming one of the main means of clinical treatment at present, and has been widely used.^[6–12]^

Ultrasound-guided percutaneous thermal ablation of thyroid nodules is a minimally invasive treatment technology that uses probe puncture to intervene in thyroid nodules under the precise guidance of ultrasound to generate heat and cause local high temperature damage to thyroid nodules. It has the characteristics of reduced trauma, improved safety and accuracy, improved treatment outcome, increased efficiency, faster recovery, fewer complications, better cosmetic appearance, and greater preservation of thyroid function after surgery so that patients do not need to take medicine for life.

Ultrasound-guided percutaneous thermal ablation of thyroid nodules has been widely used in clinical practice because of its obvious advantages and because it is highly effective.^[13–15]^ This treatment mainly includes radiofrequency ablation (RFA), microwave ablation (MWA), and laser ablation (LA). The principle of radiofrequency ablation treatment is to use the high-frequency electromagnetic wave generated by the radiofrequency instrument inserted into the thyroid nodule to make the positive and negative ions in the cell move rapidly, causing frictional heating so that the thyroid nodule will produce coagulative necrosis due to high temperature, and then be absorbed and cleared by the body’s immune system. RFA is suitable for the treatment of small nodules. The principle of microwave ablation treatment is to accurately place the microwave electrode into the nodule under the guidance of ultrasound and send electromagnetic waves through the high-speed vibration of sound waves to generate high temperature inside the nodule for a short time, which will significantly increase the temperature inside the nodule, and rapidly cause coagulative necrosis of the tissue. The necrotic tissue will be gradually cleared by the autoimmune system, and eventually the lesion will gradually shrink to disappear. The principle of laser ablation treatment is to accurately place the laser needle into the nodule, release the laser in the nodule, raise the temperature to more than 100 °C, and the heat generated will cause the tissue to carbonize, vaporize and even evaporate, and the nodule will undergo coagulative necrosis. RFA and MWA are more widely used than LA. The price of laser and of the other thermal ablation (TA) devices differs widely between different countries. In Europe, the cost of a disposable kit with a single fiber for LA ranges from 300 to 500 Euros. The cost of a disposable applicator ranges from 500 to 1000 Euros. The cost of MWA applicators is about 600 Euros in Europe. In China, the cost of LA is more expensive than RFA and MWA. Moreover, RFA of thyroid nodules performed in public hospitals is reimbursed by Chinese medical insurance, while MWA and LA is not. MWA is more suitable for the treatment of large nodules due to its high thermal efficiency. It is expected to become the preferred treatment method for benign thyroid nodules.

At present, there are still some disputes about the factors related to the efficacy of ultrasound-guided percutaneous thermal ablation in the treatment of thyroid nodules. Therefore, it is particularly important to correctly understand the factors affecting the efficacy of thermal ablation, explore reliable factors that affect the efficacy of ablation, and select appropriate and effective treatment schemes for different patients.

## 2. Influence factors of US-guided thermal ablations for benign TNs

### 2.1. The initial size and volume of the nodules

Deng and Wang^[16]^ reported that ultrasound-guided thermal ablation for the treatment of benign thyroid nodules had an obvious effect and high safety. It could not only improve aesthetic appearance, but also help patients recover after surgery. The treatment effectiveness was related to the size of the nodules. Grünwald et al^[17]^ reported that the initial volume of the nodules was an important parameter influencing therapeutic efficacy of the treatment. Liu et al^[18]^ also reported that the nodule volume was an independent factor influencing the efficacy of ultrasound-guided percutaneous thermal ablation. Xia and Hu ^[19]^ conducted a multivariate logistic regression analysis and found that the large nodule volume was an independent risk factor affecting nodule regression and absorption. The prognosis and ablation effect was better in the group with smaller nodules.

Bisceglia et al^[20]^ found that pretreatment volume > 22.4 milliliter (mL) (HR 0.54, p 0.036) was found to be an independent negative predictor of Volume reduction radio(VRR). Dai et al^[21]^ reported that there were statistically significant differences in the efficacy of different sizes of nodules from 6 to 24 months after surgery. The smaller the size of nodules, the greater the VRR after 6 months after surgery. For patients with small nodules, the scope of ablation should be expanded during operation to minimize the nodules or even completely ablate them. For patients with large nodules, the goal was to reduce the volume of nodules, in order to improve the compression symptoms and meet the aesthetic requirements of patients. After 6 months, small nodules with complete ablation obtained higher VRR than larger nodules without complete ablation.

Zhang et al^[22]^ found that the effect of ablation was due to the complete necrosis of nodules and the absorption of necrotic tissue. After ablation, the needle biopsy of the lesion showed hyperplasia of interstitial fibrous tissue with vitreous degeneration and necrosis, infiltration of macrophages, lymphocytes, and other inflammatory cells around the nodules were also present. The process of shrinking the necrotic tissue after ablation relied on the phagocytosis of inflammatory cells such as macrophages to clear the degenerated necrotic tissue. Due to the large volume of solid nodular necrotic tissue, the body needed longer time to absorb. Liu et al^[23]^ proposed that the final volume reduction of large nodules after multiple ablations was more significant than that of single ablation. Therefore, for solid nodules with large volume, the main purpose of treatment should be to reduce the tumor volume and relieve symptoms, instead of pursuing one-time complete ablation.

The larger the thyroid nodule was, the longer the nodule absorption time after ablation was. This might be because the absorption of necrotic tissue after ablation was the process of local immune response of the body. The necrotic tissue was engulfed by macrophages, lymphocytes, and other inflammatory cells. There was more necrotic tissue in the larger nodules, and the time required for phagocytic clearance was longer.

### 2.2. The gender of the patients

Sun et al^[24]^ reported that the gender of patients was an independent influencing factor of short-term efficacy after ablation. It was reported that the ablation effect of male patients was better than that of female patients.^[25]^ The level of psychological resilience of male patients with thyroid nodule ablation was higher than that of female patients, and it was an independent influencing factor that affected the postoperative outcome. Female patients also could have estrogen receptors in the thyroid tissue of female patients. Estrogen could stimulate thyroid tissue hyperplasia and promote nodule growth. However, some studies had not found the correlation between the patients’ gender and ablation efficacy. We considered that any gender differences might be related to the selective bias of the sample. On the one hand, female patients had higher risk of the disease than male patients, and the risk of thyroid nodule in female patients was about 3 times higher than that in male patients. On the other hand, female patients because of aesthetics concerns, were more inclined to treat benign thyroid nodules with minimally invasive methods such as ablation.^[26]^

### 2.3. The component of the nodules

Grünwald et al^[17]^ reported that the structure and the echogenicity were important parameters influencing therapeutic efficacy. Cao et al^[27]^ also reported that the echogenicity and the component were screened out as factors influencing VRR in benign thyroid nodules after thermal ablation in the study. Fu et al^[28]^ found that the internal component of the nodules was an independent influence factor of ablation efficacy. Liu et al^[29]^ also found that the internal component of the nodules was an independent influencing factor of short-term efficacy after ablation. Wang et al^[30]^ reported that the proportion of cystic components of nodules being <50% was an independent influence factor affecting the resorption of nodules through multivariate regression analysis. Dai et al^[21]^ reported that there were statistically significant differences in VRR between benign thyroid nodules of different natures 1 and three months after operation, of which the VRR of cystic nodules was the highest, while there was no statistically significant difference in VRR between benign thyroid nodules of different natures 6 months after operation. The reason was related to the different ablation strategies for different types of nodules. The cystic nodules needed to be aspirated and then ablated, so the VRR of the cystic nodules would be overestimated. In addition, the liquid produced more steam under cauterization, thus strengthening the process of nodule thermocoagulation, so the VRR of the cystic nodules was greater than that of other nodules 1 and 3 months after operation. However, all the nodules with different components showed obvious contraction after 6 months, and there was no statistically significant difference in VRR between the groups.

Grünwald et al^[17]^ reported that cystic nodules and mixed-pattern lesions responded slightly better than solid nodules after TA. Luo et al^[31]^ showed significantly lower VRR in solid nodules compared with those mainly solid and cystic nodules in follow-up examinations at 1, 3 and 6 months. Fu et al^[28]^ reported that for multivariate regression analysis, the cystic component was a positive prognostic factor for VRR at 1, 3, and 6 months follow ups. Kuo et al^[32]^ reported that the proportion of cystic components was independently associated with treatment efficacy in a multivariate regression analysis, and a subgroup analysis focusing on solid nodules indicated a negative correlation between echogenicity and VRR.

The efficacy of ablation was related to the nodule components, and the efficacy of ablation of cystic nodules was better, which might relate to the following 3 factors. First, during the ablation, the cystic area was first aspirated, resulting in the immediate reduction of the volume of the nodule, and then the cystic wall and the solid part of the nodules was ablated. The cystic wall of the nodule had a strong secretory function, and the ablation of the cystic wall could reduce the recurrence of the cystic-solid nodule. Second, the ablation energy required for solid and mixed nodules was higher. The greater the proportion of solid nodules were, the greater the scope and energy of ablation was needed, and the longer the overall shrinking absorption process after surgery was. Third, the more solid the component of the nodules was, the more significant cooling effect mediated by blood perfusion was, which resulted in incomplete ablation of the nodules and affected the absorption effect of the nodules after ablation.

### 2.4. The location of the nodules

The location of the nodules was a risk factor that affected the success of the treatment. The blood vessels and other structures around the thyroid are complex. The risk assessment and grading of thyroid nodule ablation can be carried out according to the position relationship between thyroid nodules and recurrent laryngeal nerve, trachea, common carotid artery, esophagus, and anterior cervical muscle group. In order to avoid thermal damage to the above important structures during the ablation process, the operator may be prone to incomplete ablation near the edge of the nodule, which will affect the postoperative effect. The ablation of the large nodules adjacent to the “dangerous triangle” and other important structures should be appropriately reserved in order to avoid thermal burns caused by excessive local heat, such as injury of the recurrent laryngeal nerve and other tissue. In multivariate regression analysis, the node close to the dangerous triangle is an independent risk factor affecting the node resorption.^[30]^ Cao et al^[27]^ reported that the enhancement defect was screened out as factors influencing VRR in benign thyroid nodules after thermal ablation.

There was statistically significant difference in the risk profile of different locations, and the effect of ablation of high-risk nodules was the worst. The nodules located in a dangerous area cannot be completely ablated in order to avoid hurting the vital tissue during the ablation treatment. Moreover, for the nodules near the common carotid artery, superior and inferior thyroid artery and other vessels, because of the blood flow in the vessels carrying away the heat in the surrounding areas (heat sink effect), the ablation area cannot rise to the expected temperature, resulting in incomplete ablation, which affects the postoperative outcome.^[33]^ Finally, the resection of nodules with abundant blood vessels surrounding it might not be very thorough, and it was easy to form a false boundary of the focus.

Wu et al^[34]^ reported that the postoperative residual nodule rate of patients with preoperative risk grading of low, medium, high, and extremely high were 1.59%,6.14%,14.43%, and 71.19% respectively. There was statistically significant difference between the low-risk group, the medium-risk group, the high-risk group and the extremely high-risk group. Zhang et al^[22]^ reported that the location of thyroid nodules adjacent to the trachea easily lead to the mirror artifacts of the trachea wall, which affected the surgical outcome. They found that the thyroid-tracheal cartilage-tracheal cavity reflection interface was conducive to the formation of mirror artifacts. Mirror artifacts mainly occurred in the acoustic image position of the tracheal cavity, which might be related to the rich gas in the tracheal cavity. The near-total reflection of air on ultrasound was the anatomical basis for the formation of mirror artifacts. However, the thyroid nodules adjacent to the trachea are prone to form the mirror reflection artifacts of the trachea wall during the ablation, which would affect the postoperative outcome. Therefore, according to the different anatomical locations of nodules, preoperative ablation risk assessment should be carried out, risk prevention and control should be needed, and effective and targeted ablation strategies such as through liquid isolation method, lever prying method and other intraoperative ablation techniques^[35]^ should be adopted to reduce the surgical risk, in order to improve the ablation effect, safety and accuracy of ablation and to reduce the complication occurrence. The liquid isolation is to inject physiological saline or sodium hyaluronate gel and other isolation solutions between the thyroid and important tissue structures such as the carotid artery, trachea, esophagus, parathyroid gland and the nerves, in order to reduce the thermal damage of important structures during ablation and relieve patients’ discomfort.^[36]^

### 2.5. Blood supply of the nodules

Wang et al^[30]^found that blood supply in thyroid nodules affected the ablation effect. They studied binary logistic regression analysis, and found that the blood supply of nodules was an independent factor of ablation efficacy. Liu^[35]^ found that the effect of nodule ablation with less blood supply was better than that of nodules with rich blood supply before operation. Dong et al^[37]^ reported that higher blood supply influenced the completed absorption of ablation (CAA) after TA (*P* = .036 and 0.003, respectively). Bini et al^[38]^ reported that a higher blood perfusion increased the heat dispersion, requiring a different combination of TA power and time treatment to achieve the target VRR. This effect might be due to the incomplete blocking of nutrient vessels in ablation of nodules with rich blood supply. The nodules with rich blood supply before operation were difficult to distinguish from the surrounding vessels, and it was also difficult to completely destroy the nutrient vessels during ablation. Therefore, the nodules with less blood supply had better VRR and lower recurrence rate than the nodules with rich blood supply. The absorption of residual nodules after ablation depended on the phagocytes in the blood. In an ideal state, the abundance of blood vessels around and inside the necrotic area after ablation affected the number and transport speed of phagocytes, and affected the absorption process. The growth of nodules depended on the nutrient vessels. Before ablation, the location and number of the nutrient vessels of nodules should be accurately determined, which should also be targeted for ablation. It was particularly important to accurately evaluate the blood supply of nodules before operation and reasonably damage the nutrient vessels during operation.

### 2.6. Hashimoto thyroiditis (HT)

HT is an autoimmune disease. Its pathogenesis is related to the destruction of immune tolerance and lymphocyte aggregation in the thyroid. Its main pathological feature is that the normal thyroid follicular structure is widely replaced by lymphocytes, plasma cells, and lymphoid germinal centers, producing various antibodies such as antioxidant enzyme antibodies.^[39,40]^ Different thyroid parenchyma backgrounds, thyroid blood flow, and hormone levels are different.

Dong et al[37] reported that chronic thyroiditis exhibited an influence on the CAA after TA (P = .036 and 0.003, respectively). Yang^[41]^ reported that the reduction rates of HT group were smaller than that of the group without HT 1 month and 3 months after the operation, and the differences were statistically significant. It was reported that HT was a risk factor affecting the regression and absorption of nodules. The thyroid is an organ prone to inflammatory changes. Zhang et al^[42]^ reported that the volume of nodules without HT decreased more significantly after radiofrequency ablation. The VRR of nodules of benign thyroid nodules after ablation was (93.2 ± 2.6)% at 18 months, which was lower than that of nodules without HT. Le et al^[43]^ also reported that the absorption of residual nodules in HT patients was slower. It may be related to the destruction of immune tolerance and the infiltration of inflammatory cells that destroy the immune response process of necrotic tissue absorption.

## 3. Prospects

With the popularization of the minimally invasive concept and the development of minimally invasive technology, the treatment of benign thyroid nodules presents a trend of diversification and humanization. As a new technology, ultrasound-guided thermal ablation of benign thyroid nodules has high clinical values and is being gradually accepted by patients. It has obvious advantages of being minimally invasive, efficient, cosmetically appealing, economical, protect thyroid function, and leaves little scar. Therefore, our next research should focus on discussing the indications, contraindications, and operation specifications of benign thyroid nodule ablation in an all-round way, in order to provide more valuable clinical basis for surgical method selection and follow-up guidance of benign thyroid nodules.

## Acknowledgments

We would like to thank Dr Wenbai Zhou (University of Southern California, USA) for proofreading this manuscript.

## Author contributions

**Conceptualization:** Liying Wang.

**Methodology:** Liying Wang.

**Project administration:** Liying Wang.

**Writing – original draft:** Liying Wang.

**Supervision:** Shusen Zheng.
